# 

**Published:** 1894-02-03

**Authors:** 


### CONTENTS.

**The Opium Problem** - - - - - - - - - 297

**Animal Temperature Under Narcotics.** By Sir BENJAMIN WARD RICHARDSON, M.D., F.R.S. - - - - - - - - - 299

**Current Medical Topics.**—Diphtheria, Scarlet Fever, and Sore Throat - - - - - - - - - - - 300

**Medical Progress and Hospital Clinics**—Acute Infective Periostitis in Children: Its Causation and Treatment - 305

Insomnia: Its Causes and Treatment - - - - - - 306

MEDICAL PROGRESS—Pulmonary Diseases—Pneumonia - - 307

Surgery of Intestines - - - - - - - - - 308

NEW DRUGS AND PREPARATIONS.—Hydrastinine and Calcium Chloride - - - - - - - - - - - - - 311

NEW APPLIANCES AND THINGS MEDICAL.—Patent "Ozonised" Digestive Tea—Food Florador—"Champetier de Ribes" Bags 312

**The Field of Science** - - - - - - - - - - 298

**The Study of Man.** IV.—Origin and Development - - 301

**Art and Disease.** IV.—Artistic Deformity - - - - 302

**Annotations.**—The "Preventive" Institute: Another Complexion—The Microbe or the Missionary?—A Life Assurance Medical Officers' Association - - - - - - - - 303

**Notes and News** - - - - - - - - - - - - 304

**The Institutional Workshop**—

HOSPITAL ADMINISTRATION—New Hospitals v. The Extension of Existing Hospitals in London - - - - - - - 313

HOSPITAL CONSTRUCTION—The Cochin Hospital, Paris - 314

PRACTICAL DEPARTMENTS—Ventilation and Ventilators - 315

HOSPITAL FESTIVALS AND MEETINGS - - - - - - 315

**Editor's Letter-Box—Scraps and Gleanings** - - - 316

### EXTRA SUPPLEMENT.—THE NURSING MIRROR.

**News from the Nursing World.**—The Children's Hospital—St. Thomas's Hospital Nurses' Concerts—A Royal Visit to Portsmouth—Trained or Untrained?—Nurses' Co-operation—The Midwives' Institute—Sunday Dinners—When Can I See the Matron?—Paupers Still Neglected—An Aristocratic Disease—A Well-organised Scheme—Short Items - - - clxxi
**On Nursing the Nervous and Insane.** IV.—Massage (*continued*) - - - - - - - - - - - - - clxxiii
**District Nursing.**—V. District Hygiene - - - - - clxxiv
**St. Michael's Home of Rest, Lyme Regis** - - - clxxv
**Nursing Classes at Bow—Sick Nurses in Italy** - clxxv
**Our Examination Questions** - - - - - - clxxvi
**The Nursing of Ovariotomy Patients** - - - - clxxvi
**Nursing in the United States.**—A New Work of Training "Attendants" - - - - - - - - - clxxvii
**Women's Work.**—A Volunteer Medical Staff Corps for Women clxxviii
**Modern Teachers.**—Mrs. Sarah Grand - - - - - clxxix
**Midwifery Training at the Bristol Royal Infirmary** clxxix

